# Supplementary material for: Plasma Exosomal microRNA Profile Reveals miRNA 148a-3p Downregulation in the Mucosal-Dominant Variant of Pemphigus Vulgaris
Source: Int J Mol Sci. 2023 Jul 15;24(14):11493. doi: 10.3390/ijms241411493 (PMC10380621; doi:10.3390/ijms241411493)
Supplement: Supplementary file 1 [file ijms-24-11493-s001.zip › ijms-2457151-supplementary.pdf]

Article

Plasma Exosomal microRNA profile reveals miRNA 148a-3p downregulation in mucosal-dominant variant of Pemphigus Vulgaris

Anna Valentino [1#], Stefania Leuci [2#], Umberto Galderisi [3], Gianrico Spagnuolo [2], Michele Davide Mignogna [2], Gianfranco Peluso [1,4*], Anna Calarco [1,4]

[1] Research Institute on Terrestrial Ecosystems (IRET)—CNR, Via Pietro Castellino 111, 80131 Naples, Italy; anna.valentino@cnr.it (A.V.); anna.calarco@cnr.it (A.C.).
[2] Department of Neurosciences, Reproductive and Odontostomatological Sciences, Oral Medicine Unit, Federico II University of Naples, 80138 Naples, Italy. stefania.leuci@unina.it (S.L.), gianrinco.spagnuolo@unina.it (G.S.), mignogna@unina.it (MD.M.).
[3] Department of Experimental Medicine, University of Campania "Luigi Vanvitelli", Via Santa Maria di Costantinopoli, 80100 Naples, Italy. umberto.galderisi@unicampania.it (U.G.).
[4] Faculty of Medicine and Surgery, Saint Camillus International University of Health Sciences, Via di Sant'Alessandro 8, 00131 Rome, Italy. gianfranco.peluso@unicamillus.org (G.P.).
* Correspondence: gianfranco.peluso@unicamillus.org (G.P.)
# These authors contributed equally to this work.

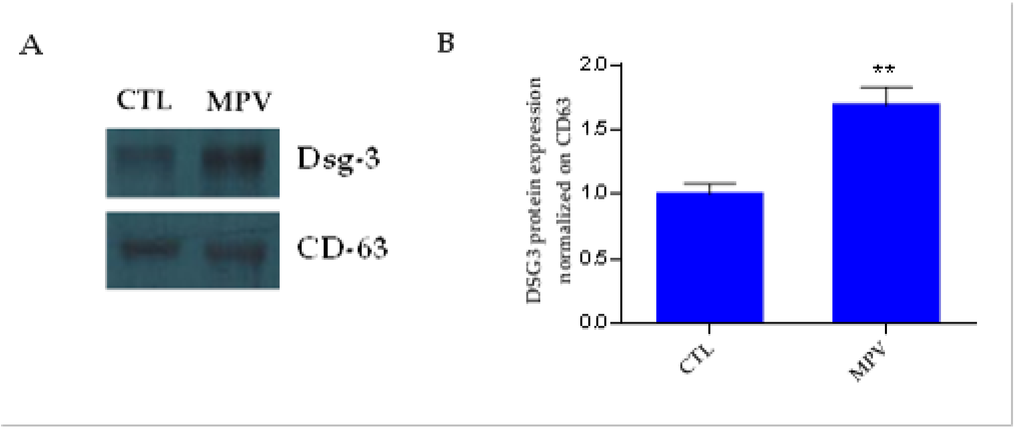

Figure S1. Western blot analysis of plasma derived-exosomal protein. (**A**) Dsg-3 exosomal protein expression in MPV plasma samples respect to healthy subjects. CD-63 was used as control protein. (**B**) The protein levels were quantified by ImageJ. The bars represent the means ±S.D, (n =6). Statistically significant variations **p<0.01 versus CTL.

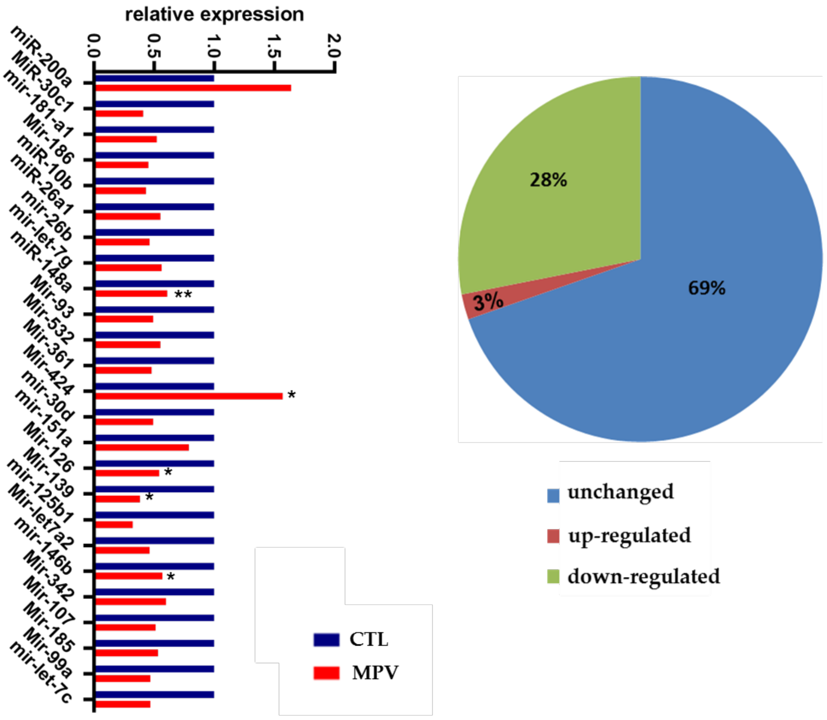

Figure S2: Exosomal miRNAs expression profile in MPV. Statistically significant variations ** $p<0.001$ * $p<0.05$ versus CTL.

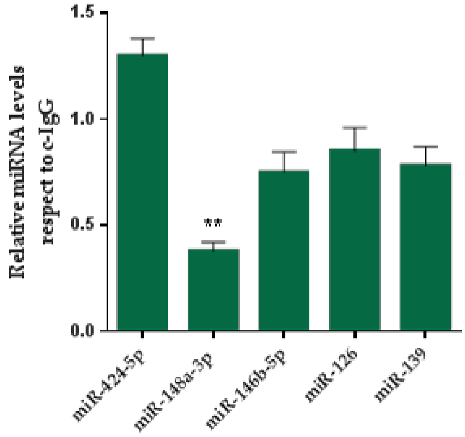

Figure S3. HEKa cells miRNA expression levels. MiR-424-5p, miR-148a-3p, miR-146b-5p, miR-126 and miR139 expression level derived from HEKa cells treated with MPV-IgG respect to c-IgG determined by TaqMan-RT-PCR assay. Statistically significant variations ***p*<0.01 versus c-IgG.

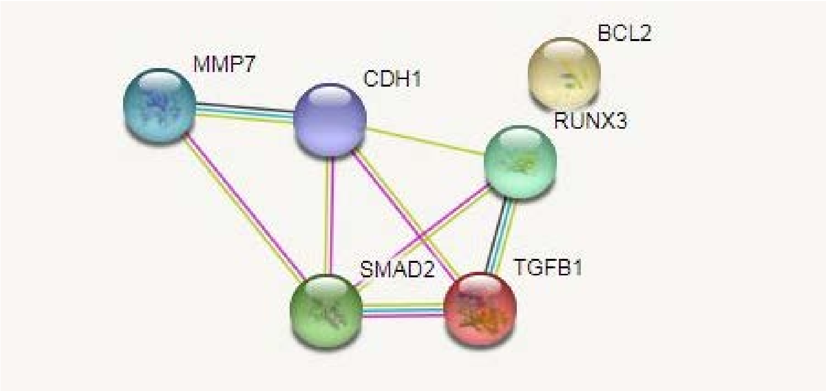

Figure S4: STRING Gene Ontology enrichment analysis of differential expressed proteins (Version 11.5). An intricate protein-protein interaction network among the regulated proteins by miR-148-3p.
